# Astrocyte Differentiation of Human Pluripotent Stem Cells: New Tools for Neurological Disorder Research

**DOI:** 10.3389/fncel.2016.00215

**Published:** 2016-09-26

**Authors:** Abinaya Chandrasekaran, Hasan X. Avci, Marcel Leist, Julianna Kobolák, Andras Dinnyés

**Affiliations:** ^1^BioTalentum LtdGödöllő, Hungary; ^2^Department of Medical Chemistry, University of SzegedSzeged, Hungary; ^3^Dorenkamp-Zbinden Chair, Faculty of Mathematics and Sciences, University of KonstanzKonstanz, Germany; ^4^Molecular Animal Biotechnology Laboratory, Szent Istvan UniversityGödöllő, Hungary

**Keywords:** astrocyte, glial, central nervous system (CNS), Alzheimer disease (AD), brain pathology, microglia, CNTF, brain damage and repair

## Abstract

Astrocytes have a central role in brain development and function, and so have gained increasing attention over the past two decades. Consequently, our knowledge about their origin, differentiation and function has increased significantly, with new research showing that astrocytes cultured alone or co-cultured with neurons have the potential to improve our understanding of various central nervous system diseases, such as amyotrophic lateral sclerosis, Alzheimer’s disease, or Alexander disease. The generation of astrocytes derived from pluripotent stem cells (PSCs) opens up a new area for studying neurologic diseases *in vitro*; these models could be exploited to identify and validate potential drugs by detecting adverse effects in the early stages of drug development. However, as it is now known that a range of astrocyte populations exist in the brain, it will be important *in vitro* to develop standardized protocols for the *in vitro* generation of astrocyte subsets with defined maturity status and phenotypic properties. This will then open new possibilities for co-cultures with neurons and the generation of neural organoids for research purposes. The aim of this review article is to compare and summarize the currently available protocols and their strategies to generate human astrocytes from PSCs. Furthermore, we discuss the potential role of human-induced PSCs derived astrocytes in disease modeling.

## Introduction

Central nervous system neurons are never alone; they are often connected with astrocytes along with other cell types to form structural and functional networks. Astrocytes are the most abundant cell types in the CNS (Azevedo et al., 2009) with a remarkable heterogeneity both in morphology and function. In the past, astrocytes were believed to act as “passive support cells” for electrically active neurons and to be primarily responsible for cellular homeostasis of the CNS, but current research shows their active participation in many other processes such as the formation of neural networks, recycling of neurotransmitters, and detoxification (Nedergaard et al., 2003; Krencik and Ullian, 2013). Many other functions are also beginning to emerge as the research on astrocytes continues, and our understanding of their disease-relevant cellular functions in several diseases has already been revised. Here, we review the role of astrocytes, compare their *in vivo* and *in vitro* differentiation, and discuss the pathomechanisms of certain diseases in which they are involved.

## The Role of Astrocytes in the CNS

Astrocytes play a direct and critical role in the developing CNS in maintaining an optimal environment for the normal development and function of neurons. Some examples of astrocytic functions include energy supply, the formation of the BBB, and removal of toxins and debris (described below). Impairments in these functions, as well as physiological fluctuation in glutamate/K^+^ levels, can trigger or exacerbate neuronal dysfunction (Zhang et al., 2016). Based on their important and physiological role, it is not at all surprising that changes in astrocytes can directly affect the behavior of rodents (Franke and Kittner, 2001).

### Energy Supplies for Neurons

One of the oldest known functions of astrocytes is to supply energy in the form of lactate to neurons. Glucose is mainly stored as glycogen in astrocytes, where it is metabolized to pyruvate and lactate and then transported via MCTs across the cell membrane. The transported lactate is then utilized by neighboring neurons and metabolized (Magistretti et al., 1999). Apart from glucose metabolism, astrocytes are also involved in glutamate uptake via two pathways. The first pathway involves the direct conversion of glutamate to α-ketoglutarate through NAD-dependent oxidative deamination catalyzed by GDH, and the second pathway is an ATP-requiring reaction in which ammonium is catalyzed by GS to yield glutamine. This glutamate-glutamine shuttle protects against the toxic effects caused by extracellular glutamate (Sonnewald et al., 1997).

### Maintenance of the Cellular Homeostasis of the Brain

One essential function of astrocytes is to maintain brain homeostasis through multiple dynamic equilibrium adjustments, including water balance, ion distribution, glutamate buffering, and recycling (Wang and Qin, 2010; Coulter and Eid, 2012). High levels of synaptic glutamate can cause over-activation of neurons which may lead to excitotoxicity; thus rapid removal of extracellular glutamate from the synaptic cleft is essential for neuronal survival (Dong et al., 2009). This is accomplished by Na^+^ dependent transporters on astrocytes, EAAT1 and EAAT2, respectively.

Apart from glutamate clearance, astrocytes can control cerebral glutamate levels (Stobart and Anderson, 2013). Glutamate that is taken up by the astrocytes is converted to glutamine by GS, then later passed back to the synaptic terminal where it is converted back to glutamate (Danbolt, 2001; Parpura and Verkhratsky, 2012). There is increasing evidence that the uptake of glutamate also induces glycolysis in astrocytes, resulting in the production and secretion of lactate for the neighboring neurons (Ricci et al., 2009; Bélanger et al., 2011; Stobart and Anderson, 2013). This mechanism, the astrocyte to neuron lactate shuttle, regulates lactate delivery in an activity-dependent manner (Pellerin et al., 1998; Stobart and Anderson, 2013).

### Formation and Maintenance of the Blood–Brain Barrier

Together with endothelial cells and pericytes of the brain microvessels, astrocytes form the BBB, a physical diffusion barrier which restricts the exchange of most molecules between blood and brain (Abbott et al., 2006; Macvicar and Newman, 2015). Astrocytes are also involved in regulating cerebral blood flow by a K^+^ siphoning mechanism, releasing K^+^ onto blood vessels from their end-feet in response to neuronal activity (Paulson and Newman, 1987). It has been suggested that the release of prostaglandins from astrocytes results in increased Ca^2+^ that evokes vessel dilation (Zonta et al., 2003). Likewise, they are also involved in regulating BBB permeability from the bloodstream to brain parenchyma by the activation of tight junction proteins through NF-κB (Brown et al., 2003; Abbott et al., 2006). BBB defects are involved in many neuroinflammatory and neurodegenerative diseases, including multiple sclerosis, where the specialized brain endothelial cells which comprise the BBB are diminished, causing a loss of protective function during the progressive phase of disease (Weiss et al., 2009).

### Synapse Formation, Maintenance, and Pruning

There is now abundant evidence to support the notion that astrocytes are actively involved in the formation and refinement of neural networks (Oberheim et al., 2006; Araque and Navarrete, 2010). During development, billions of neurons connect to form functional networks via synapses, with the control of synapse development by astrocytes highly conserved across species. A distinctive attribute of astrocytes in synapse formation is to increase the number of synaptic structures (dendritic spine) within the neural circuits (Ullian et al., 2001; Slezak and Pfrieger, 2003; Stevens et al., 2007; Stipursky et al., 2011; Clarke and Barres, 2013). The first evidence for astrocytes being involved in synapse formation came from the rodent RGC study, which showed that culture with astrocytes resulted in a 10-fold increase in excitatory synapse and synaptic functionality (Meyer-Franke et al., 1995). Later, *in vitro* studies confirmed that astrocytes can also instruct synapse formation for human neurons (Diniz et al., 2012).

Astrocytes are also involved in the refinement of the neural network by synaptic pruning - the elimination of extra synapses to increase the precision and efficiency of neural circuits (Clarke and Barres, 2013). The mouse retinogeniculate system, an excellent model system for studying synapse refinement and elimination (Hong and Chen, 2011), has been used to show that signals released from astrocytes in the postnatal brain induced the expression of the complement component C1q that executes synapse elimination by astrocytes via phagocytosis (Stevens et al., 2007). Notably, astrocytes employ this mechanism throughout the nervous system (e.g., in the uninjured brain or in response to glioma or trauma). However, further work is required to investigate the phagocytic pathway of astrocytes in human models.

### Communication between Astrocytes and Neurons

Studying the direct communication between astrocytes and neurons is a rapidly expanding field of neuroscience. The term “tripartite synapse,” was proposed 20 years ago to describe synaptic physiology involving astrocytes, in addition to pre- and post-synaptic neurons. In this context, astrocytes release neuroactive molecules (such as glutamate, ATP, nitric oxide, prostaglandins, and D-serine) in the presence of elevated Ca^2+^, which in turn influence neuronal excitability (Araque et al., 1999; Perea et al., 2009; Eroglu and Barres, 2010).

The concept of “gliotransmission,” which was first hypothesized in the 1980s, involves the active vesicular release of neurotransmitters and glutamate by astrocytes (Halassa et al., 2007). In other words, the GPCR-mediated Ca^2+^ variations in astrocytes can trigger the release of glutamate, D-serine, and ATP. So far, various mechanisms have been proposed for gliotransmission, including Ca^2+^-regulated vesicular exocytosis (Agulhon et al., 2012) and non-vesicular release. Additionally, Santello et al. (2011) found that cytokines are required for functional glutamatergic gliotransmission. While the importance of gliotransmission is supported by findings that dysfunction of astrocytic proteins involved in transmitter release can cause severe brain disorders (Rossi et al., 2011), there are still several features of gliotransmission that are controversial and require further clarification (Hamilton and Attwell, 2010; Sahlender et al., 2014; Sloan and Barres, 2014).

## Classification of Astrocytes: A Heterogenic Group of Cells

Astrocytes can exist in two distinct developmental stages: a highly proliferative state, which occurs within the developing brain in the first weeks after birth, or a mature state/post-mitotic astrocytes (Ge et al., 2012). The peculiar morphology of mature mammalian astrocytes was first observed in mice by Otto Deiters in 1865 using chromic acid and carmine red staining (Deiters and Guillery, 2013). A detailed morphological study achieved by Camillo Golgi and Ramón y Cajal, who developed the black staining reaction and produced drawings of stained glial cells in 1872, revealed the distinct morphological pattern of the protoplasmic and fibrous astrocytes. These cells were first called “astrocyte” in 1893 by Mihály Lenhossék, a Hungarian anatomist and histologist, who stated that astrocytes are a mixed population of cells and not a single cell type. Based on our current understanding, there are a range of astrocyte subtypes differing in their cellular morphologies and anatomical locations in the brain (summarized in **Table 1**). Astrocyte morphologies vary considerably amongst cortical regions and display distinct biochemical/biophysical properties throughout discrete regions of the cortex (Emsley and Macklis, 2006; Regan et al., 2007). Due to their diversity, developing a full characterization of astrocytes is challenging.

**Table 1:** Classification of astrocytes.

Types of astrocytes	Anatomical locations	Cellular morphologies	Functions	Reference
Protoplasmic astrocytes	Grey matter	Short branched;	• Maintenance of the blood–brain barrier;	Peters et al., 1991; Bushong et al., 2002; Nishiyama et al., 2002; Ogata and Kosaka, 2002; Oberheim et al., 2006; Sofroniew and Vinters, 2010
		Thick processes	• Regulation of blood flow;	
			• In synapse formation;
			• Neuronal metabolism

Fibrous astrocytes	White matter	Thin and straight processes	• Maintenance of the blood–brain barrier;	Sofroniew and Vinters, 2010
			• Regulation of blood flow;
			• In synapse formation;
			• Neuronal metabolism

Interlaminar astrocytes	Pial surface (humans and monkey)	Spherical cell bodies	• Regulation of calcium wave;	Oberheim et al., 2009
			• Thick network of GFAP fibers

Varicose projection astrocytes	Fifth and sixth layer of the cerebral cortex	Long processes. (up to 1 mm in length)	Unknown	Oberheim et al., 2009, 2012

Epithelial glial cells (Bergmann glia)	Purkinje-cell layer of cerebellum	Long processes	• Synaptic transmission.	Fuller and Burger, 1992; Olude et al., 2015

Fañanas cells	Cerebellar cortex	Feather-like arrangement	Unknown	Fuller and Burger, 1992

Müller cells	Retina of juvenile	A type of radial glial	• Retinal homeostasis;	Fuller and Burger, 1992; Vecino et al., 2015
			• Phagocytosis of neural debris;
			• Metabolic activity;
			• Glycogen within their cytoplasm

Pituicytes	Neurohypophysis	Irregular shaped cytoplasm	Unknown	Burger and Scheithauer, 1993

Interstitial epiphyseal cells	Epiphysis	Cytoplasmic processes	Unknown	Fuller and Burger, 1992


*Astrocytic domain organization varies with pathology and anatomical locations.*

## Identification of Astrocytes

The identification of astrocytes *in vivo* is usually based on staining of the cells for their content of GFAP. This microfilament protein shows a high specificity for astrocytes in brain tissue. As it is expressed in virtually all reactive astrocytes, it is particularly useful for identification of astrocytes in diseased brain. On the basis of such studies, it is known that the cells can change their morphology if they are activated or form scars (Anderson et al., 2016), but in general they display a typically stellate morphology. Rodent studies have shown that GFAP expression is not essential for astrocytes, and that subpopulations of resting astrocytes do not express the microfilament protein (Kuegler et al., 2012). As also subpopulations of resting human astrocytes may not stain for GFAP, their morphology is yet little characterized, and new markers are urgently needed (Zhang et al., 2016). The capacity of astrocytes to change shape is also obvious *in vitro*. If pure populations are cultured under standard conditions, cells are found to be flat and of a roughly polygonal or feathery shape, but if astrocytes are co-cultured with neurons, they can assume a stellate shape. Whilst most of the studies on astrocyte cultures were based on the rodent, Guillemin et al. (1997) isolated and characterized astrocytes from primate brain to study the differences between human and non-human primate CNS. Later it was confirmed that *in vivo* primate astrocytes have a stellate morphology, and express high levels of GFAP (Oberheim et al., 2009). Further, it was found that transient acidification of the culture media resulted in stellation of cultured primate astrocytes, accompanied by an increased expression of GFAP and vimentin (Renner et al., 2013). Although the non-human primate brain is an important model system essential for studying the primate brain, it is important to test whether the results can be generalized to the human brain, especially in the context of human diseases. Some of the markers that start to appear in both *in vitro* and *in vivo* are described below. The first indication of the glial specification is marked by induction of nuclear factor NFIAA/B and GLAST (Araque and Navarrete, 2010) which appears in embryonic development at E11.5 in the mouse. Both of these markers remain expressed during glial precursor migration. Furthermore, GLAST is also expressed in the RG cells. Other markers such as S100β, FGFR3, FABP7, BLBP, and SOX9 (which are required for the neuron-glial switch) are not exclusive, astrocyte-committed markers but are expressed during neurogenic stages. For instance, while S100β is an astrocyte progenitor marker, it is also expressed in oligodendrocytes progenitors (Deloulme et al., 2004; Hachem et al., 2005). A fascinating issue in this field is how to identify a mature astrocyte and how to standardize this definition worldwide, and two laboratories are notable for their work in this area (Krencik et al., 2011; Roybon et al., 2013). One of the hallmarks of astrocyte identification is GFAP, the major interfilamentous protein of a mature astrocyte (Fox et al., 2004). However, while the expression pattern of GFAP is a sensitive, reliable marker for most of the reactive astrocytes that respond to CNS injuries, additional markers such as AQP-4, GS, GLT-1, and GLAST-1 should also be used to study astrocyte differentiation (Kuegler et al., 2010; Krencik and Zhang, 2011; Krencik and Ullian, 2013; Kleiderman et al., 2016). A new astrocytic early stage marker, ALDH1, that selectively labels cortical astrocytes *in vivo* has recently been discovered (Rowitch and Kriegstein, 2010) *in vivo*, and ALDH1A1 expression has been demonstrated to serve as a reliable marker in early astrocytic differentiation (Alexandra Adam et al., 2012). **Table 2** summarizes our current knowledge of astrocyte-specific markers in both human and mouse. For instance, during early development, immature astrocytes express mainly vimentin while at the end of gestation period vimentin is replaced by GFAP in differentiated astroglial cells. However, additional studies are still needed to clarify the complex roles of astrocytes.

**Table 2:** List of human and mouse astrocyte markers.

Marker Name	Role/Localization	Reference
Aldolase C (Fructose-bisphosphate aldolase C)	Targets glycolysis	Li et al., 2013; Roybon et al., 2013

Aquaporin-4	Perivascular membranes of astrocytes	Gee and Keller, 2005; Kuegler et al., 2010; Roybon et al., 2013; Kleiderman et al., 2016

A2B5 (c-series ganglioside-specific antigen)	Astrocyte precursors and type 2 astrocytes	Dietrich et al., 2002; Kuegler et al., 2010; Hayashi et al., 2011; Kleiderman et al., 2016

Aldehyde dehydrogenase family 1 member L1	ALDH1L1 and GLT1 are co-expressed in the same population of astrocytes; Appears at mid-embryogenesis	Cahoy et al., 2008; Rowitch and Kriegstein, 2010; Roybon et al., 2013

Bystin	Stains reactive astrocytes	Sheng et al., 2004

Connexin 43	Astrocyte specific marker in the human brain	Lovatt et al., 2007; Fatemi et al., 2008

Glial fibrillary acid protein	Low GFAP at a quiescent state and High GFAP in mature state	Ghandour et al., 1980; Rodnight et al., 1997; Kuegler et al., 2010; Roybon et al., 2013; Kleiderman et al., 2016

GLT-1^∗^	Early astrocytes; Postnatal marker	Chaudhry et al., 1995; Kuegler et al., 2010; Roybon et al., 2013; Kleiderman et al., 2016

GLAST 1	Mature astrocytes; Appears at mid-embryogenesis	Chaudhry et al., 1995; Kuegler et al., 2010; Roybon et al., 2013; Kleiderman et al., 2016

Nuclear factor 1A	Gliogenic switch	Roybon et al., 2013

Nestin	Reactive glial cells	Classic marker

Glycogen phosphorylase	Astrocyte specific marker in the human brain	Pfeiffer et al., 1992

S100β	Early astrocyte marker	Burette et al., 1998; Carlén et al., 2009; Kuegler et al., 2010; Kleiderman et al., 2016.

Vimentin	Canonical marker	Deneen et al., 2006; Roybon et al., 2013



*Based on the available data, this simplified table shows the identified astroglial markers in current trend. The onset of expression varies in different regions of the CNS. ^∗^ in rodents: Excitatory amino acid transporter 2.*

Astrocytogenesis can also occur in neurogenic hot spots, correlated with the production of new neurons. In adult rodent brain, neurogenesis occurs in the SVZ of the lateral ventricle and the subgranular zone (SGZ) of the hippocampal dentate gyrus (Ma et al., 2009). Observations that cells with astrocytic markers (GFAP and S100β; Zhang and Jiao, 2015) begin to emerge in the granular cell layers of the dentate gyrus (Doetsch et al., 1997; Kriegstein and Gotz, 2003) are providing new insights into adult neurogenesis. Moreover, inflammatory and pathological changes may result in the conversion of astrocytes to neural stem cells. Whether this also applies to the human brain requires further investigation (Robel et al., 2011; Dimou and Götz, 2014; Götz et al., 2015).

## Origin of Astrocytes

Gliogenesis generally follows neurogenesis in the developing brain. Our knowledge on “origin and lineage progression of human RG” has mainly been extrapolated from rodent studies due to the limited access of human brain tissue, but recent studies have begun to uncover unique structural and cellular features of the primate brain (Duan et al., 2015). The human neocortex contains around 16 billion neurons of diverse subtypes (Lui et al., 2011). As the neuroepithelial cells expand the cortical plate, they form an elongated bipolar cell type, RG cells (Ming and Song, 2005). Transformation of neuroepithelial cells into RG emerges at the beginning of neurogenesis and occurs in humans over the course of months, while the process only takes “days” in the rodent brain (Pollen et al., 2015). RG can be distinguished from neuroepithelial progenitors by the expression of astroglial markers.

### Radial Glial Division

**FIGURE 1:**
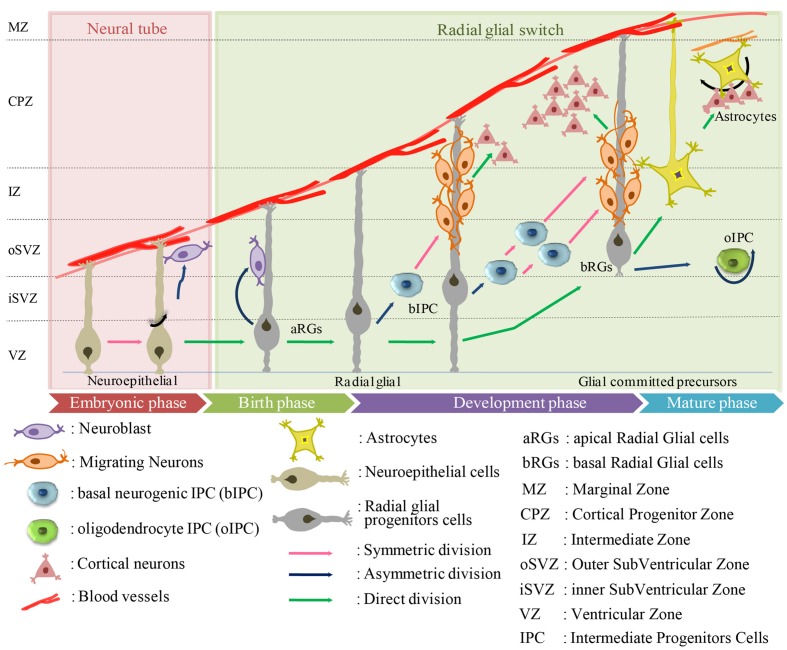
**Origin of human astrocytes from the developing neuroectodermal tube toward mature phase.** The neuroectodermal cells give rise to ependymoblasts which either differentiate into astrocytes or glial membrane on the external side of the neural tube.

Radial glial are the earliest cells to be derived from the lateral wall of the neural tube (Anthony et al., 2004; Howard et al., 2006; Mo et al., 2007) (**Figure 1**). In humans, RG cells are also found in the oSVZ in the developing neocortex, which is absent in rodents (Pollen et al., 2015). The oSVZ contains a large proportion of outer RG (oRG) cells that act as guides for neuronal migration. Particularly in humans, the oRG cells appear to contribute to neocortical expansion by increasing the number of neural precursor cells (NPCs) (LaMonica et al., 2013).

Radial glial cells usually divide at the apical (ventricular) surface within the ventricular zone (VZ) to generate new neurons or single IPC by asymmetric division, or self-amplify progenitors by symmetric division. The majority of these progenitors migrate radially with the rapid increase in the width of the cerebral wall (Hartfuss et al., 2001; Marín and Rubenstein, 2001; Anthony et al., 2004; Götz and Huttner, 2005; Sild and Ruthazer, 2011; Florio and Huttner, 2014; Schitine et al., 2015). Just before birth, the RGs “accelerate the expansion of the neuronal population” and switch to gliogenesis to produce astrocytes. This transition from neurogenesis to astrogenesis is mediated by known soluble factors including IL-6 and BMP-4 protein (Miller and Gauthier, 2007). By the end of the cortical development, most of the RG cells lose their ventricular attachments and migrate toward the cortical plate to form different cortical layers.

In general, there is a remarkable overlap in the expression patterns and cell proliferative processes of human and rodent, but there are species differences in structural organization and complexity. In mice, the majority of cells form a single compact layer, whereas in humans they are dispersed throughout a larger zone. IPCs in human VZ regions can be divided into two subpopulations, apical and basal, that have discrete molecular profiles. Apical IPCs are defined by mitosis occurring at the ventricular surface and the basal IPCs are defined by mitosis occurring at an aventricular location and the absence of ventricular contact (Florio and Huttner, 2014). In general, IPCs can generate neurons (nIPCs) or glial cells, including oligodendrocytes (oIPCs) or astrocytes (aIPCs). Mouse IPC go through only one round of cell division to produce two neurons (LaMonica et al., 2013), whereas human IPC undergo several rounds of cell division before producing neurons (Hansen et al., 2010). The molecular mechanisms that underlie IPC division are still not clear but their transcriptional regulators, such as TBR2, EOMES are likely involved (Kowalczyk et al., 2009; Fietz et al., 2010; Hansen et al., 2010). Many reports have suggested that SVZ in humans can be subdivided into the iSVZ and oSVZ (Hansen et al., 2010; Lewitus et al., 2013; Thomsen et al., 2016). A new type of outer radial glia-like cells, basal RGs (bRGs), was identified in the oSVZ of developing cortices of humans (Betizeau et al., 2013; Pilz et al., 2013). The translocation of RG cells and differentiation to astrocytes has been visualized through time-lapse imaging (Noctor et al., 2008), as well as a similar transformation in the sub-cortical telencephalon (Barry and McDermott, 2005). To date, five different sources of cortical astrocytes have been identified: (i) RG cells within the ventricular zone; (ii) RG cell transformation; (iii) intermediate progenitors; (iv) glial progenitors in marginal zone; and (v) superficial layer progenitors. However, only a limited number of studies on astrogliogenesis have been performed in the human (Kanski et al., 2014; Paşca et al., 2015), in contrast to the numerous reports on rodents (Chi et al., 2006; Ventura and Goldman, 2007; Widestrand et al., 2007; Demars et al., 2010; Ge et al., 2012).

## Molecular Triggers and Regulators

Before moving on to consider human astrocyte development *in vitro* to generate authentic human astrocytes, we must first consider the *in vivo* mechanism.

Inside a human cortex, each astrocyte can be connected to several thousand neurons to form the neural network. For instance, each astrocyte can associate with the neural process to form many neuronal synapses (2,000,000 in humans) (Oberheim et al., 2009). Astrogenesis is mainly initiated by the activation of JAK-STAT, the canonical pathway regulating astrocyte gene expression (Bonni et al., 1997), although multiple signaling pathways participate (Wen et al., 2009). For example, STAT3 signaling is crucial for astrogenesis, and STAT3 activation requires the presence of the p300/CBP co-activator complex to initiate astrocyte gene expression (Freeman, 2010). STAT3 is activated by tyrosine kinases belonging to the Src and JAK families. This occurs as a consequence of cytokine or growth factor receptor activation and has been shown to be relevant for the EGF receptor (epidermal growth factor receptor), the GCSF (granulocyte colony stimulating factor) receptor or the IL-6 and CNTF receptors (Moon et al., 2002; Freeman, 2010). In addition to STAT3, the Notch pathway is another important regulator of cell fate. Notch activation directly regulates the *HES* family of the *bHLH* gene, inhibiting neurogenesis during the neurogenic period and promoting astrogenesis during the gliogenic period (Kageyama et al., 2005, 2008). Other signaling pathways, such as BMP-SMAD, and Nuclear factor IA (NFIA) can also promote astrogenesis in the presence of an active JAK-STAT signal (Bonni et al., 1997; Nakashima et al., 2001; Deneen et al., 2006; Nagao et al., 2007; Nakanishi et al., 2007; Stipursky et al., 2012). Even though the mechanisms underlying mammalian astrocyte commitment have not fully been characterized, this work has shed light on the essential signaling pathways that are responsible for the transition.

A combination of several molecules such as TGF-alpha, CNTF, LIF, IL-6 cytokines, and oncostatin M are required for astrocyte stimulation *in vivo*. Other factors such as BMPs are also involved in determining astrocytic fate. For instance, a delay or disruption in any of the signaling pathways can hinder the epigenetic mechanism and timing of neurogenesis and astrogenesis, eventually leading to perturbations in the relative ratios of the cell types (Yan et al., 2005; Tidyman and Rauen, 2009).

### Generation of Human Astrocyte from Pluripotent Stem Cells

Examination of human astrocytes from post-mortem tissue have led to a better understanding of brain diseases and opened doors toward generating more efficient *in vitro* based models. The first human astrocytes were cultured from fetal or adult post-mortem tissue (Ennas et al., 1992; Lee et al., 1993), but were often contaminated with microglia and other cell types which were difficult to separate during dissections. Additionally, in many cases, biopsies represent the end stage of the disease and control tissue is obviously inaccessible due to ethical concerns and potential health risks. Given all the practical limitations of human brain tissue research, murine and rat astrocytes have mostly been used to study astrocyte physiology. For this purpose, murine or rat astrocytes are usually purified from the cortex. Alternatively, astrocytes may be generated from murine PSCs (Kleiderman et al., 2016). These generalized approaches do not account for regional heterogeneity of astrocytes, such as the expression of the transporter OCT3, which is high in striatal astrocytes and low in cortical astrocytes (Cui et al., 2009). Especially for disease studies, astrocytes may therefore be purified from the relevant brain regions. For instance, cerebellar, cortical, striatal, and nigral astrocytes show differences in dopamine or angiotensin signaling (Yu et al., 1996; Reuss and Unsicker, 2000). Thus, astrocytes from specific brain regions offer an attractive alternative source to study astrocyte function *in vitro*. Mice models offer further advantages since methods to manipulate the genome (knock-out or knock-in genes) are well-established. With these approaches it needs, however, to be considered that astrocyte heterogeneity may not be a cell-intrinsic property, but be decided by the surrounding neurons, and may therefore get lost in cell culture (Farmer et al., 2016).

#### Comparison of Human and Rodent Astrocytes

The long list of interspecies variation between human and rodent astrocytes underlines the need for authentic human astrocytes for disease modeling. Importantly, there are several visible differences between rodent and human astrocytes:

##### (i) The average length of astrocytes

Human astrocytes are structurally more complex than mouse astrocytes (Oberheim et al., 2006, 2009). An investigation of the total arborization length of mouse and human astrocytes *in vitro* found that the average astrocyte process in human was almost twice as long as that in rodents *in vitro* (Zhang et al., 2016).

##### (ii) Average branch numbers

There is a difference in the average branch number *in vitro* for humans and rat: 8.5 ± 1.1 and 4.5 ± 0.5, respectively (Zhang et al., 2016). This dataset was consistent with *in vivo* measurements (Oberheim et al., 2009).

##### (iii) Glial to neuron ration

A much higher ratio of glia to neurons has been estimated for the human cortex (∼1.65:1) than for rodent (∼0.3:1) (Nedergaard et al., 2003; Sherwood et al., 2006).

##### (iv) Different classes of GFAP positive cells

There are only two main types of astrocytes in mouse: fibrous astrocytes and protoplasmic astrocytes. In addition to these broad classes, two additional subtypes have been identified in human and other primates: interlaminar and varicose projection astrocytes (Colombo et al., 1995; Reisin and Colombo, 2004) (**Table 1**).

##### (v) Gene expression pattern

Only about 90% of the expressed genes in mouse and human astrocytes overlap (Sun et al., 2013), so there is the opportunity for unique sets of genes to up-regulate or down-regulate during astroglial development (Zhang et al., 2014, 2016). Differences have been found in the glutamate response (Zhang et al., 2016), and in the use of the TLR/IL-1R receptor and immune activation. For instance in mouse astrocytes, LPS induced mostly an A1 effective response, thereby producing abundant IL-1 protein. In the case of human astrocytes TLR4 receptor complex proteins and MD2 are expressed but not CD14 (Tarassishin et al., 2014). These results have critical implications for translational research of human CNS diseases.

##### (vi) Supporting these observations

Supporting these observations, essential differences *in vivo* between the two species include *in vivo* speed of calcium signaling, which is five times faster in humans (Sun et al., 2013), and the number of neuronal synapse networks, from 1 × 10^4^ in mouse to up to 2 × 10^6^ in humans (Oberheim et al., 2012). *In vitro*, adult human astrocytes responded differently to extracellular glutamate levels than those of adult mouse, which remained quiescent under the same conditions (Zhang et al., 2016).

Most strikingly, various drugs that showed promise in an animal model have failed in human trials (Cavanaugh et al., 2014; Cummings et al., 2014). Therefore advancement toward more human-relevant models is critical for the study of neurological disorders.

To overcome these obstacles, various laboratories have elaborated *in vitro* differentiation protocols to generate astrocytes from hPSCs. In the early 2000s, hESCs held great promise and were considered to be the most reliable source for the generation of human astrocytes and many other neuronal cell types. However, despite their potential benefits in disease modeling, the controversial and ethical issue of their derivation from early embryos remains. Takahashi and Yamanaka (2006) and Takahashi et al. (2007) published a groundbreaking method for generating iPSCs using four transcription factor (TF) genes (*Oct4, Sox2, Klf4*, and *c-Myc*) to reprogram somatic cells into PSCs. The discovery opened new possibilities in stem cell research providing new and ethically acceptable cell sources for PSC generation, and making it possible to derive stem cells directly from patients with different diseases, such as neurological disorders.

Since the advent of hiPSC technology, several groups have developed differentiation protocols to obtain human astrocytes from various pluripotent cell sources (hESCs and hiPSCs; see details in **Table 3**), very often adapted from protocols in published studies (**Table 3**). These protocols are continuously being upgraded to improve efficiency and functionality and differ significantly in multiple aspects (listed in **Table 3**), such as the cell seeding density at plating, the substrate, media composition, the timing and concentration of exogenous growth factors and morphogens, and the physical dimensions of the culture system (monolayer or embryoid bodies). These differences which might seem small at first glance make it very complicated to compare the outcome of the different methods. In this section, we aim to examine some of the most commonly used techniques in human astrocyte differentiation to provide a point of reference (also summarized in **Table 3**).

**Table 3:** Current protocols for astroglial differentiation of human PSCs.

Reference	Cell source	Method of Differentiation	Key players	Research Outcome	Early Markers	Mature/Late Markers
Zhang et al., 2001	hESCs	EB	FGF-2: 20 ng/ml cAMP: 100 ng/ml BDNF: 10 ng/ml PDGF-A: 2 ng/ml	GFAP+ both *in vitro* and *in vivo*	Nestin, Musashi-1, PSA-NCAM	NF200, GFAP

Carpenter et al., 2001	hESCs	EB	RA: 10 μM hEGF: 10 ng/ml hbFGF: 10 ng/ml hPDGF-AA: 1 ng/ml hIGF-1: 1 ng/ml hNT-3: 10 ng/ml hBDNF: 10 ng/ml	–	Nestin, PSA-NCAM, A2B5, MAP-2, Synaptophysin	

	mESCs	NS		–	Vimentin, NF1A, GLAST, ALDH1L1, GLT-1	GFAP, AQP4, S100β

Tabar et al., 2005	hESCs	ML	FGF2: 20 ng/ml EGF: 20 ng/ml Noggin: 500 ng/ml SB 431542	At fourth week, 2% expressed astrocyte marker	Nestin, calretinin, DLX2, NCAM, A2B5	β-III -Tubulin, EGFAP

Itsykson et al., 2005	hESCs	EB		Glial fate observed at 25th week	GABA, glutamate, serotonin, tyrosine hydroxylase, O4	GFAP, β-III-Tubulin,

Johnson et al., 2007	hESCs	NS	Heparin: 2 μg/ml FGF2: 20 ng/ml BDNF: 10 ng/ml GDNF: 10 ng/ml cAMP: 1 μM Ascorbic acid: 200 μM	By nineth week astrocyte appeared in the neural network	For synaptic analysis MAP2, Synapsin-1, β-III-Tubulin,	GFAP, S100β

Hu et al., 2010	hESCs and iPSC	EB	**Glial:** RA: 100 nM SHH: 100 ng/ml cAMP: 1 μM **Oligodendrocytes:** PDGF-AA: 60 ng/ml Neurotrophin 3: 10 ng/ml IGF1: 10 ng/ml	GFAP+ cells after 3 months and excitatory postsynaptic currents were observed in >8 weeks culture (but efficiency unknown)	β-III -Tubulin, S100β	GFAP

Krencik., 2011, 2012	hESCs and iPSC	EB	RA: 0.5 μm FGF8: 50 ng/ml SHH: 500 ng/ml EGF and FGF2: 10 ng/ml CNTF: 10 ng/ml LIF: 10 ng/ml	Uniform populations of immature astrocytes (>90% S100β+ and GFAP+).	For synaptic analysis MAP2, Synapsin-1, β-III-Tubulin,	GFAP, S100β

Hayashi et al., 2011	rat iPSC	NS	FGF-2: 20 ng/ml FBS: 10%	NSC differentiated exclusively into astrocytes when FGF-2 was removed from neurobasal medium	Nestin, β-III-Tubulin	GFAP, S100β

Emdad et al., 2012	hESC and hiPSC	EB	SB43152: 10 μM Noggin: 500 ng/ml	55–70% of GFAP+ cells at week 5	Nestin, GLT-1, A2B5, β-III-Tubulin	GFAP, GLAST, aquaporin-4

Juopperi et al., 2012	hiPSC	EB	bFGF: 20 ng/ml	S100β and GFAP+ cells after 2–3 months (efficiency unknown)	Nestin, β-III-Tubulin, MAP2ab, doublecortin (DCX)	GFAP, S100β

Lafaille et al., 2012	hiPSC	EB	EGF/FGF2: 20 ng/ml SonicC25II: 125 ng/ml FGF8: 100 ng/ml BDNF: 20 ng/ml Ascorbic acid: 0.2 mM	90% GFAP+ cells after 60–90 days	Nestin, β-III-Tubulin	GFAP

Serio et al., 2013	hiPSC	EZS/ NS	EGF/FGF2: 20 ng/ml CNTF: 10 μg/ml	After ∼8 weeks, 90% cells positive for GFAP	Vimentin, nuclear factor 1A	GFAP, S100β

Shaltouki et al., 2013	hESC and iPSC	EB	bFGF: 20 ng/ml CNTF: 5 ng/ml BMP: 10 ng/ml bFGF: 8 ng/ml Activin A: 10 ng/ml Heregulin 1β: 10 ng/ml IGFI: 200 ng/ml	60–80% of GFAP positive cells after 5 weeks (starting from NSC).	β-III -Tubulin	GFAP, S100β

Roybon et al., 2013	mESC; hESC hiPSC	ML	LDN193189: 0.2 μM SB431542: 10 μM Ascorbic acid: 0.4 μg/ml RA: 1 μM BDNF: 10 ng/ml GDNF: 10 ng/ml	After 80 days ∼100% cells positive for S100β and ∼70% GFAP-expressing cells.	GFAP, A2B5,	GLAST, GLT1, Cx43, S100β, ALDH1L1, aldolase C
			CNTF: 10 ng/ml IGF: 10 ng/ml SHH-C: 200 ng/ml 1% FBS bFGF: 20 ng/ml			

Sareen et al., 2014	hiPSC	EZS	EGF: 100 ng/ml FGF2: 100 ng/ml Heparin: 5 μg/ml RA: 0.5 μM	Increased GFAP^+^ cells.	A2B5, Aldh1L1, GFAP	S100β, AQP4, GLAST

Mormone et al., 2014	hiPSC	EB	FGF2: 10 ng/ml EGF: 20 ng/ml FGF+EGF+CNTF: 20 ng/ml Noggin: 500 ng/ml	99% GFAP+ cell population after 28–35 days	Musashi, Nestin, A2B5	GFAP, A2B5

Caiazzo et al., 2014	hfibroblast	Direct reprogramming		–	SOX9, Vimentin	GFAP

Zhou et al., 2016	hiPSC	EB	LDN193189: 0.2 μM SB431542: 10 μM AA: 0.2 mM	Spontaneous emergence approach: By ∼4 weeks, GFAP+ cells were quantified.	For synaptic analysis MAP2, Synapsin-1, β-III-Tubulin	GFAP, AQP4


*There are two major approaches for differentiation of human PSCs to astrocytes: EB, monolayer culture. For both EB and monolayer approaches, stage-specific application of key growth factors (GFs) in defined media are required for optimal astrogenesis. Some protocols use fetal bovine serum (FBS) or small molecules to induce differentiation (see text for details of specific protocols). EB, embryoid body; h, human; m, mouse; AA, ascorbic acid; RA, retinoic acid; EZS, EZ spheres; ML, monolayer; NS, neurospheres. EZ spheres are similar to embryoid bodies that are passaged using tissue chopper into 200 μm spheres.*

To establish a reproducible platform and to study astrocyte-neuronal interaction, protocols first have to generate stable human NPC populations. During organismal development, the fate of the respective cell types is determined by the exact timing and concentration of growth factor/patterning signals at given locations. With knowledge of the patterning signals, *in vitro* astrocytogenesis of defined subpopulations could be achieved by exposing human PSC-derived primitive neuroepithelia to a set of diffusible signaling molecules, directing their differentiation into subpopulations that would arise *in vivo* in discrete regions along the neural tube. This process could generate functionally diversified classes of glial cells. A similar approach is commonly used for neurons (Kirkeby et al., 2012). For instance, FGF and RA determine rostro-caudal identity, whereas Wnts, BMPs, and Shh are required to specify NPCs along the dorso-ventral axis. We know that astroglial progenitors generated in the absence of mitogens carry a dorsal-anterior identity by expressing Otx2 but not Hoxb4 or Nkx2.1, while astroglial progenitors generated in the presence of RA express Hoxb4 but not Otx2 (Liu and Zhang, 2011). To mimic the *in vivo* mechanisms governing early neurogenesis (NPC formation), two major classes of protocols are utilized: an EB-based technique (with or without SMAD inhibition) and a monolayer-based dual SMAD inhibition method. Most protocols use aggregation of cells into EBs, and only a few rely on a monolayer-based adherent culture system (Shi et al., 2012; Roybon et al., 2013). The 3D aggregation system is thought to maintain the “stemness” of stem cells and to allow better cell-to-cell and cell-to-matrix interactions. Other vital considerations for improved neural cultures are media composition, exogenous growth factors or small molecules, and most importantly the timing of the procedure itself.

### The Neural Induction Protocol

**FIGURE 2:**
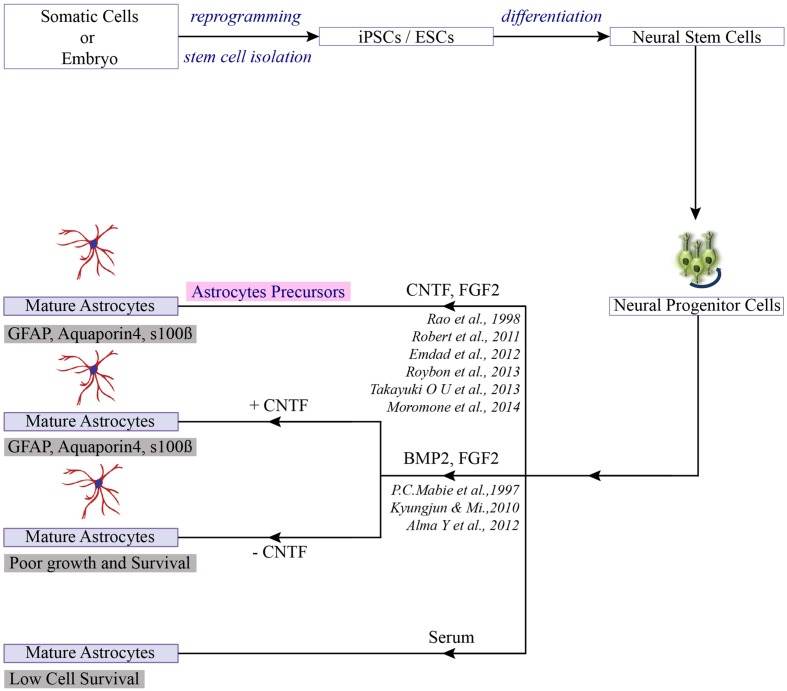
**Differentiation of PSCs toward astrocytes.** Once the neuro-progenitors are produced from pluripotent stem cells, addition of selection factors can lead to the generation of mature astrocytes or astrocytes precursor (Jha et al., 2015).

The neural induction protocol involves in dissociating PSCs and plating them on a feeder or feeder-free adherent culture system. The media for neural induction usually consist of the neurobasal medium or DMEM/F12 medium, or combination of both. The cells are rapidly induced with antagonists, either LDN or noggin to inhibit the BMP pathway and SB431542 to inhibit the TGF-ß pathway, along with additional components to enhance neuronal precursors (Chambers et al., 2009) (**Figure 2**). The primitive neuroectodermal aggregates (3D system) or neuroepithelial sheets (2D system) are then plated on the adherent substrate to promote the definitive neuroectoderm fate. Upon reaching the “end phase,” NPCs are organized into polarized structures called neural rosettes. These neural rosettes are selected and cultured for several passages and then directed toward astroglial progenitors with different combinations of morphogens (CNTF, SHH, FGF, and RA) in defined culture medium. Numerous studies have utilized each of these methods, often with minor variations. However, it is not always clear why a particular method was chosen, and so it is very difficult to evaluate the exact effect of these small changes. In the next section, we have summarized the most efficient ways to generate human astrocytes from PSC-derived NPCs through *in vitro* culture, and discuss the maturity of the derived astrocytes.

For neural differentiation of hESCs, most of the available methods are based on either the initial formation of embryoid bodies or on co-culture with stromal cells. First, we would like to compare the aggregate differentiation from monolayer differentiation.

### hESCs Studies on Embryoid Bodies

Zhang et al. (2001) was the first to develop a novel technique to isolate and culture human astrocytes in serum-free conditions, thus recapitulating the resting, non-reactive state normally observed in healthy astrocytes *in vivo*, and went on to describe a differentiation technique for human ES cells that can be applied to model and study fetal and mature astrocytes in health and disease. In their first report (Zhang et al., 2001), differentiation was initiated by culturing the cells as 3D spheres in chemically defined medium containing FGF-2 for a short period. Subsequently, the EBs were grown in adherent culture to form neural rosettes. This unique cross-sectional arrangement of epithelial cells is now considered a hallmark of successful neural induction. By day 7, almost all EBs generated neural tube structures to produce neural progenitor cells and neural stem cells, and morphological analysis confirmed the expression of neural markers Nestin, Musashi-1 and neural cell adhesion molecule (NCAM). This group were the first to show the incorporated hESC-derived neural precursors in different brain regions upon transplantation into the neonatal mouse, with no teratoma formation. This remarkable development generated GFAP^+^ astrocytes, oligodendrocytes both *in vitro* and *in vivo.*

In the same year, two other EB based differentiation studies were published (Carpenter et al., 2001; Reubinoff et al., 2001). Both studies also used hESCs in a 3D aggregate culture system in the presence of FGF-2 to induce neural differentiation. In one, EBs were differentiated into neuronal progenitors in the presence of RA along with selective morphogens (Carpenter et al., 2001) (detailed in **Table 3**), while in the other, differentiation occurred in the presence of just EGF and FGF-2 (Reubinoff et al., 2001). Therefore both the studies obtained populations with a wide panel of morphological characteristics, such as positive for Nestin, PS-NCAM, and A2B5. FACS analysis revealed 96% of the cells were positive for A2B5 (a marker for astroglial progenitors) (Carpenter et al., 2001).

### hESCs Studies on Monolayer

Later, Tabar generated a neural progenitor population by inducing hESCs on stromal cells (MS5) in serum-free media (Tabar et al., 2005). To enhance the neural induction noggin was added to the N2 media. After a month neural precursors were isolated and maintained in N2 media containing FGF-2 and EGF. At this stage, the majority of cells (>90%) were immunoreactive for neural precursor markers (Nestin, Musashi-1) and a glial progenitor marker (A2B5).

**FIGURE 3:**
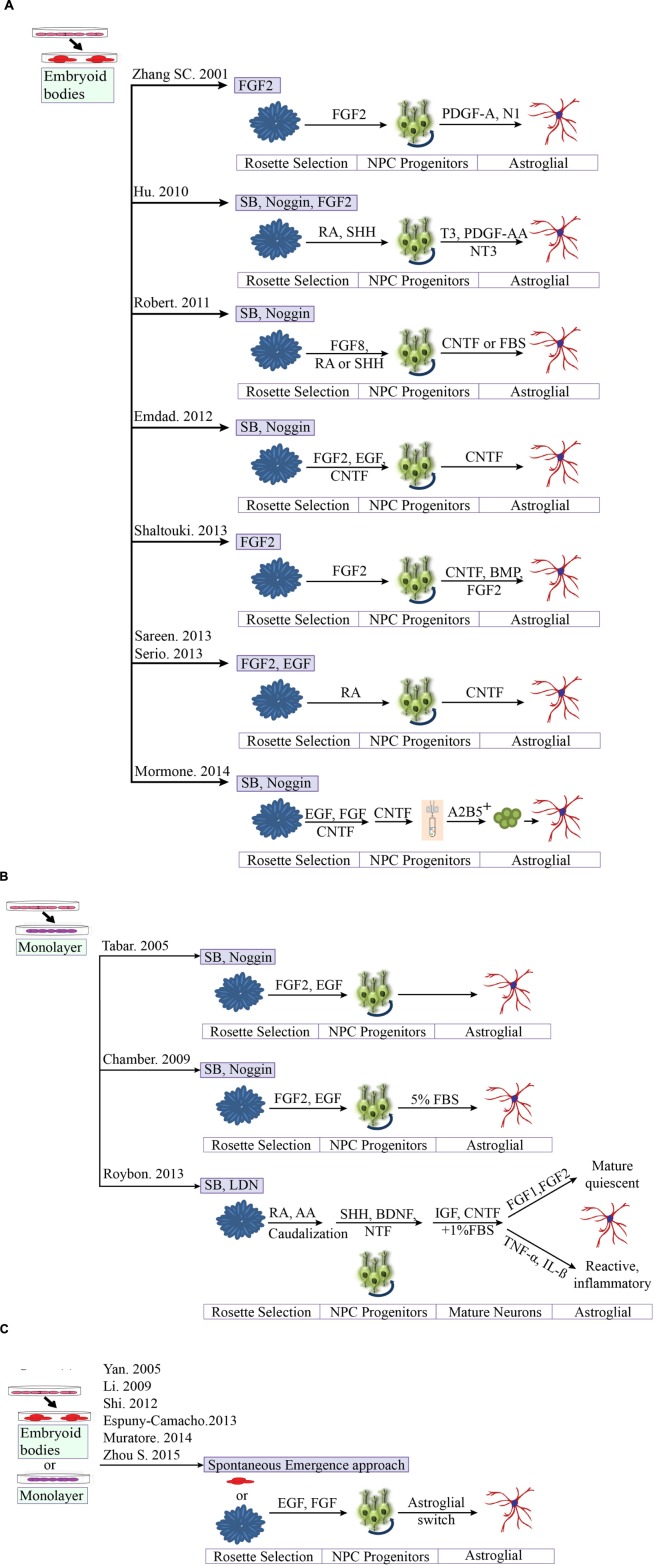
**Comparison of different protocols for deriving astroglial cells from human pluripotent stem cells.** **(A)** Generation of astroglial cells from 3D aggregates or neurosphere through neural progenitor’s cell stage. iPSCs are dissociated at day 1 and cultured as aggregates in suspension. Aggregates are replated onto culture dish to form primitive neuroepithelial structures. Upon reaching definitive NE structures are manually isolated and replated for progenitors. **(B)** Methods based on neuronal rosette selection. Rosette structures are mechanically harvested and replated in appropriate growth factors for specific progenitors **(C)** Spontaneous emergence approach. Endogenous astrocytes gradually emerge over time, say after 40 days.

A better understanding of differentiation protocols has led us to conclude that the majority of hESC studies utilize the classic aggregation method to yield functional astrocytes in combination with different growth factor cocktails. Despite the potential benefits of hESC technology, the direct differentiation of hESCs toward astrocytes is rarely undertaken, for ethical reasons. Therefore we now compare some of the available protocols from human iPSCs technology (detailed in **Table 3** and **Figures 3A,B**). For neural differentiation of hiPSCs, most methods available are either based on the initial formation of embryoid bodies or monolayer base methods.

### hiPSCs Studies on Embryoid Bodies

Differentiation of hiPSC-derived NSC into astrocytes was first reported following the observation that astrocyte progenitor cells were S100β positive after four weeks of differentiation and GFAP positive around the 12th week of differentiation (Hu et al., 2010). The study used a similar aggregated differentiation method to that of Zhang et al. (2001) to obtain neurons. To further enhance the glial differentiation the progenitors were expanded in N2B27 media containing additional factors, i.e., cAMP, T3, platelet-derived growth factor (PDGF), Insulin-like growth factors (IGFs), and neurotrophin-3 (NT-3), with the aim of increasing the formation of OLIG2 positive ventral progenitors and reducing the formation of HB9^+^ postmitotic motoneurons (Hu et al., 2010).

In the subsequent years, additional studies have described the generation of astroglial cells from human PSCs, including a landmark study that generated functional astrocytes from iPSC that were indistinguishable from hESC-derived astrocytes (Emdad et al., 2012). The study involved culturing EBs on low attachment plates in neurobasal media for 2 weeks to develop columnar epithelium within the formed EBs. And also investigates the astrocyte differentiation potential with three different conditions: (1) CT-1 alone, (2) CT-1 in combination with CNTF, and (3) recombinant Jagged-1 (JAG1-Fc) in conjunction with CNTF. The results have showed high number of hiPSC-derived NE cells after sequential treatment with FGF- 2 + CNTF followed by CNTF alone. However, no incremental effect on astrocytic differentiation was observed when CT-1, Notch activator JAG1-Fc was used.

Later, Lafaille described the efficient generation of astroglial cells within 10 weeks (Lafaille et al., 2012), adapting previously described protocols to enhance neural differentiation (Zhang et al., 2001; Elkabetz et al., 2008). The derived neural crest stem cells (NCSc) and NPCs from hPSCs were expanded in defined conditioned medium supplemented with EGF and FGF-2 for 8 weeks, followed by 5% FBS treatment for 20 days, leading to 90% GFAP^+^ cells.

The next study went on to show the efficient generation of astroglial cells within the short time frame of 6 weeks from hPSC-derived NSCs, using a defined medium system (Shaltouki et al., 2013). As might be expected, the differentiation protocol was EB based, with the colonies cultured in suspension for 8 days before surface attachment. Formed neural rosettes were manually isolated and replated to acquire a homogenous population of NSCs, that was then stimulated with neurotrophic factor (CNTF) and proteins (BMP, FGF-2) to significantly increase the proportion of GFAP positive cells. These differentiated astrocytes were then plated on neurons to quantify synapse formation. Interestingly, the number of synaptic puncta remained significantly higher in the presence of astrocytes, indicating that these *in vitro* astrocytes displayed similar functional characteristics and morphological patterns to primary astrocytes.

Other studies have added further evidence for the generation of astroglial cells. Sareen et al. (2014) generated so-called “EZ spheres” from hiPSCs that could be differentiated into neural progenitor cells with an astroglial predisposition. The “EZ sphere” is a free-floating cell aggregate containing pre-rosette stem cells, that is generated directly from PSCs. The EZ spheres were caudalized using all-*trans*-RA in neurobasal medium and maintained their proliferative capacity for 30 passages. For astroglial differentiation, they were dissociated and plated as single cells (Sareen et al., 2014). Like Sareen, the study by Serio et al. (2013) opted for EZ sphere formation; neurospheres were mechanically chopped at the beginning of the enrichment phase and cultured in neurobasal medium for 2–4 weeks, before being dissociated to form NPCs. Astrocyte populations were obtained by differentiating NPCs in CNTF (Serio et al., 2013). The resulting population robustly expressed both developmental (Nestin, vimentin, GFAP) and mature astrocyte markers (S100β).

The most striking feature of these studies is the formation of the EZ sphere (∼200 μm) which represents the primitive type of neural stem cells at a stage before early neural tube formation. While various protocols exist for the generation of PSC-derived neural progenitor cells, including the complex four stage systems involving RA-mediated induction, adherent culture, and neural rosette isolation, all these methods can be laborious when compared to EZ sphere formation.

Mormone and co-workers then generated iPSC-derived astrocytes with characteristics similar to those differentiated from hESCs by culturing clusters of cells in low attachment plates in the presence of neurobasal media supplemented with different factors such as FGF-2, EGF, and CNTF, at various time points. Cells were then subjected to immunopurification via magnetic cell sorting for the “positive selection of A2B5 cells,” before being plated in neurobasal medium containing CNTF for an additional 2 weeks. A2B5 is mostly expressed in embryonic neural tissue and is therefore considered to be a marker for immature glial-committed precursors that give rise to glial types such as astrocytes and oligodendrocytes. The authors reported 99% GFAP-positive cells at fourth week of differentiation with the absence of teratoma formation in long-term experiments (Mormone et al., 2014).

### hiPSCs Studies on Monolayer Base Methods

Roybon et al. (2013) generated two subpopulations of human astrocytes *in vitro*: the first subpopulation was generated from stem cells (derived from human PSCs) and was capable of an immunological response similar to human primary astrocytes, while the second subpopulation contained mature, quiescent astrocytes (Roybon et al., 2013). When co-cultured with MNs, these astrocytes also enhanced neuronal survival and neurite outgrowth. Strikingly, they also found that the addition of either FGF1 or FGF2 was sufficient to promote transitions, maturation/quiescence without triggering inflammatory responses (TNFα or IL-1). It should be noted that although the majority of the existing protocols administer FGF2 into the media (**Table 3**), the exact role of FGF2 in *in vitro* astrocyte differentiation is uncertain. There is evidence to suggest that FGF-2 has an additional feature in gliogenesis beyond driving the propagation of neural stem cells both *in vitro* and *in vivo*. The possible mechanisms of action are: (i) FGF2 in combination with a second factor (e.g., CNTF or BMP2), but not FGF2 alone, may trigger astrocytogenesis; (ii) FGF2 may trigger early commitment of NSC to astrocytes, but is not sufficient to transform the cells to astrocytes; or (iii) astrocytogenesis may be a stressful process for NSC, and the presence of FGF2 keeps the cells alive and proliferative, while other factors could decide the way of the differentiation (Song and Ghosh, 2004; Kang and Song, 2010).

### Emerging Trends in Astroglial Differentiation

The strategy to recapitulate neurogenesis and astrogenesis during early development has gained increased attention in modern science. The generation of human RG-type cells from PSCs in a chemically defined medium without adding morphogens validates that cultured hRGs maintain a cell-intrinsic clock that regulates the progressive generation of stage-specific neuronal and glial subtypes (Duan et al., 2015). One step further, a 3D culture approach for generating human cortical spheroids (hCSs), a laminated cerebral cortex-like structure, from PSCs initially showed only a few GFAP positive cells after 35 days of differentiation. This later increased to 8% on the 76th day and almost 20% after 180th day (Paşca et al., 2015). Finally, Caiazzo et al. (2014) demonstrated that the direct conversion of human fibroblast by overexpressing a defined group of TFs (NFIA, NFIB, and SOX9) led to the rapid single-step generation of human induced astrocytes. The study makes use of inducible TFs which are selectively expressed in astrocytes. Unlike any other studies, this research indicated a direct reprogramming process with no intermediate states resembling either ESCs or NSCs. To our knowledge, this is the first report that shows astrocyte-like cells derived from fibroblasts by entirely skipping the iPSC generation. The expression of astrocyte marker proteins (S100β and GFAP) was detected in induced astrocytes. However, further experiments are required to prove the functional similarity to human astrocytes (Caiazzo et al., 2014). Therefore, regardless of approach, it is a matter of generating authentic RG-like NSC/NPCs which can be further differentiated into astroglial-like cells. To obtain a more gliogenic NSCs/NPC type, it is, therefore, necessary to induce the differentiation with RA and subsequently immune-isolate for CD133-positive cells. This principle was shown for human cells (Gorris et al., 2015) and murine cells (Kleiderman et al., 2016).

### Astrocyte Maturation Studies

Astrocyte maturation occurs through a series of complex events, which remain poorly understood. Some reports have suggested that IL-6 family cytokines (such as LIF, CNTF) (Bonni et al., 1997) or BMPs (BMP2, BMP4) (Gross et al., 1996) have a comparable potential to induce differentiation of astrocytes from neural progenitor cells. Some published protocols have achieved astroglial maturation by culturing cells in the presence of different combinations of FGF-2, EGF, and CNTF for changing time periods (Emdad et al., 2012). Others have achieved astrocyte maturation by any of the following combinations: (i) maintenance media along with 10% fetal bovine serum (FBS) (Palm et al., 2015); (ii) maintenance media in the presence of BMP-4 (preferably for rodents); (iii) maintenance media with CNTF; or (iv) maintenance medium with bFGF, NT3 and/or PDGF-AA (Juopperi et al., 2012; Haas and Fischer, 2013; Tanga et al., 2013). These methods can generate robust populations of functionally diversified astrocytes with high efficiency, based on staining for β-III-tubulin, GFAP, and AQP4, but their functional maturation and efficacy varies considerably among different studies and cell lines.

### Spontaneous Emergence Approach

Astrocytes can also emerge spontaneously in neuronal cultures by the “spontaneous emergence approach” (**Figure 3C**). For example, human iPSC-derived NPCs have been differentiated toward various neuronal lineages such as cortical neurons (Li et al., 2009), midbrain dopaminergic neurons (Yan et al., 2005) and spinal MNs, where astrocytes and oligodendrocytes frequently appear as side products in these preparations, however, the efficiency is low and not reliable. A numbers of protocols have been derived from this approach. A recent study showed the emergence of endogenous astrocytes from neuronal cultures via increased expression of the glial fibrillary acidic protein (GFAP), a marker of astrocytes, at day 100, with confirmation through the NanoString platform (Muratore et al., 2014). Other groups have also confirmed the emergence of astrocytes with long-term differentiation of hiPSCs-derived neural progenitors (Shi et al., 2012; Espuny-Camacho et al., 2013). A new 3D based protocol published by our group was able to generate neuronal cultures enriched in GFAP^+^ and Aquaporin-4^+^ astrocytes (Zhou et al., 2016). However, all of these protocols are complex, time-consuming, difficult to expand and have fewer functional properties than the *in vivo* models or directed approach.

### Toward a Standardization of Protocols

The generation of astrocytes from human PSC has reached a state where the cells can be considered for clinical applications and various research questions. It is now important to take stock, to identify remaining research needs and to work toward protocols that provide standardized sets of cells. Concerning the latter point, it would be important to develop consensus criteria of how a ‘high quality astrocyte population’ should be defined. Such criteria will need to account for different applications of the astrocytes. For instance, the requirements for cells intended for transplantation will be different from those required for pre-clinical target discovery, or disease modeling, or construction of *in vitro* 3D tissues. However, in all instances, the cell population will need to be characterized for: (i) purity according to positive markers and also for negative markers; (ii) for maturity/region-selectivity, e.g., by using markers, secretion of growth factors and/or gene expression profiling; (iii) function, e.g., by functional assays like looking at glutamine synthesis or inflammatory activation or by quantifying the support of neuronal growth and synapse formation; as well as (iv) cell cycle state - most of the cells should be non-proliferative. To our knowledge, none of the available protocols has been fully characterized for all this information, and each available protocol still has drawbacks: (i) some of the protocols do not provide sufficient detail for reproduction; (ii) astrocyte purity and maturity is not comparable to that obtained with primary isolated astrocytes; (iii) there are technical issues that prohibit widespread use, even though the protocol may be suitable (e.g., long-term induction process that might result in >120 days protocol to generate astrocytes); and (iv) the emerging astrocytes are often poorly characterized (e.g., are only stained for classic markers such as GFAP and S100β), but hardly for maturity markers, regional identity, negative markers and additional astrocyte functional characterization. Some astrocytes have been generated from PSC in the presence of patterning molecules known to shape the nervous system. In this way either spinal cord phenotypes (Krencik et al., 2011; Roybon et al., 2013; Serio et al., 2013; Sareen et al., 2014) or astrocytes with forebrain identity have been generated (Shaltouki et al., 2013). However, most studies have not explored the region-specific phenotype of the astrocytes generated *in vitro*.

For this reason, there is no protocol at present that has become a gold standard, and there is still a need both for further protocol optimization and for better characterization and standardization of the resultant cell types.

## Astrocytes in Neurological Diseases

Since the importance of astrocytes for functional neuronal networks has long been underestimated, it is not surprising that their central role in many neurological disorders was equally neglected. Experiments on mouse models of human neurological diseases including diverse neurodegenerative diseases (e.g., AD, ALS, Parkinson’s disease, and spinocerebellar ataxia) and neurodevelopmental disorders (e.g., Alexander’s disease, Autism spectrum disorders, Epilepsy and Rhett syndrome) have led to advances in understanding astrocyte biology (**Table 4**, for the overview of pathological affected gene defects). In most of the diseases, however, it remains unclear whether the pathologic changes are caused by astrocytes or rather a consequence of preceding events.

**Table 4:** Summary of the astrocyte-affected diseases.

Astrocyte affected disease	Astrocyte associated defects or gene defects	Astrocytes mediated pathological or neurological features	Reference
Hepatic encephalopathy	Astrocyte swelling and neurotransmitter receptor alternation	Cytotoxic brain oedema	Felipo and Butterworth, 2002; Butterworth, 2010; Chen et al., 2012; Takayama, 2015

Neuromyelitis optica (NMO)	Auto-antibody; loss of aquaporin 4^+^ and GFAP^+^ astrocytes	Inflammation, demyelination, astrocyte loss and nerve injury	Lennon et al., 2004; Wingerchuk et al., 2007; Marignier et al., 2010; Tradtrantip et al., 2012

Balo’s disease	Loss of aquaporin4^+^ and CX43	Demyelination and astrocyte hypertrophy	Tokumoto et al., 2010; Czepiel et al., 2011; Pouya et al., 2011; Linnoila and Chitnis, 2014

Wernicke’s encephalopathy	Loss of EAAT1 (GALST) and EAAT2 (GLT-1); decreased level of aquaporin 4	Loss of neurons, oedema, gliosis	Sechi and Serra, 2007; Lough, 2012

Alexander disease (AxD)	Mutation in GFAP protein	Accumulations of eosinophilic cytoplasmic inclusions, known as RF	Prust et al., 2011; Wang L. et al., 2011; Molofsk et al., 2012; Lanciotti et al., 2013

Alzheimer disease (AD)	Synaptic loss and neuronal death	Deposition of extracellular SPs and neurofibrillary tangles	Wyss-Coray et al., 2003; Koistinaho et al., 2004; Castellano et al., 2011; Li et al., 2011; Sidoryk-Wegrzynowicz et al., 2011; Israel et al., 2012; Kondo et al., 2013

Vanishing white matter (VWM)	Autosomal recessive neurological disease, Mutation in eIF2B, eIF2B is a guanine nucleotide-exchange factor for eIF2	Gray matter remains normal, whereas white matter changes texture. Abnormally shaped astrocytes with febrile infections	La Piana et al., 2012; Elroy-Stein and Schiffmann, 2014

Megalencephalic leukodystrophy	Is an early-onset macrocephaly, Degeneration of motor functions	Intramyelinic vacuole formation, alterations of the blood–brain barrier structure and astroglial activation	Anand et al., 2007; Brignone et al., 2014



*Aldh1L1, aldehyde dehydrogenase 1.*

It is worthwhile to mention the success of iPSC technology in regards to neural-glial disease modeling including Parkinson disease (Rhee et al., 2011; Sánchez-Danés et al., 2012), demyelination (Tokumoto et al., 2010; Czepiel et al., 2011; Pouya et al., 2011), retinal regeneration (Parameswaran et al., 2010; Tucker et al., 2011), nerve degeneration (Wang A. et al., 2011) and various others (Saporta et al., 2011). Recent studies by Chen et al. (2014) shed light on understanding the disease phenotypes of DS using an iPSC tool. This study tested minocycline (an FDA approved drug) to correct the pathological phenotypes of DS astroglia. Notably their results demonstrated higher levels of GFAP/S100β expression as compared to control astroglia. Therefore iPSCs technologies are now considered to be a valuable platform to model diseases and improve our understanding of pathomechanisms. Furthermore, they can be employed for drug screening and testing. In the following section, we would like to discuss some of the diseases that could see, or already have seen, benefits from this technology.

### Alexander Disease (AxD)

Alexander disease is a progressive astrogliopathy caused by a dominant gain-of-function mutation in the *GFAP* gene that maps to chromosome 17q21 (Brenner et al., 2009). It is a primary disease of astrocytes that affects neural development and causes mental retardation, seizures, and megaencephaly in early childhood. There are three age-dependent clinical subtypes of AxD: infantile, juvenile and adult-onset. Infantile AxD is very aggressive and is characterized by seizures, and bulbar dysfunction with low life expectancy. Juvenile AxD is characterized by hyperreflexia and bulbar symptoms affecting children between 2 and 14 years of age, and is milder than the infantile form. Adult AxD which affects the late adolescence and beyond, has similar characteristics to the juvenile form (Prust et al., 2011).

In this disease, there is no known metabolic abnormality. Pathophysiology reveals abundant astrocytic accumulations of eosinophilic cytoplasmic inclusions, known as RF, one of the hallmarks of AxD (Molofsk et al., 2012). RF are protein aggregates composed of Vimentin, GFAP and small heat shock proteins that are present in the cytoplasm of astrocytes. The clinical signs of AxD are likely due to astrocyte dysfunction rather than astrocyte loss (Messing and Goldman, 2003). *In vitro* studies have provided additional clues that astrocyte-mediated effects in AxD are due to oxidative stress and reduction in the GLT-1. Therefore these observations suggest a possible pathogenic mechanism underlying neuronal loss in AxD. Other possible astrocytic failures that contribute to myelin degeneration in AxD are K^+^ buffering, and Na^+^/K^+^ATPase activity (Lanciotti et al., 2013). To address the clinical importance of astrocyte dysfunction in AxD, there is a critical need for the new model system, as so far only a Drosophila model exists (Wang L. et al., 2011).

### Amyotrophic Lateral Sclerosis (ALS)

Amyotrophic lateral sclerosis is an adult-onset neurodegenerative disease manifested by degeneration of MNs in the motor cortex, brain stem, and spinal cord, resulting in muscle paralysis and ultimately motor neuronal death (Rowland and Shneider, 2001; Kiernan et al., 2011). Most of the cases of ALS are sporadic with unknown genetic mutation; only about 5–10% of ALS cases are caused by known genetic mutations, among which 20% are caused by mutations in the copper/zinc SOD-1 gene. Mutations of SOD1 were identified through an autosomal-dormant inheritance pattern (Rosen et al., 1993). Gurney et al. (1994) were the first to recapitulate the hallmarks of ALS using a transgenic mouse model (SOD1 G93A mutant mouse) that expresses human copies of SOD1 enzyme with glycine-alanine substitution at the 93rd codon of the coding sequence (Gurney et al., 1994; Rothstein et al., 1995). Over the years, a variation of SOD1 mutants have been subsequently developed as G37R, G85R, and G86R transgenic; although these models differ in protein content, they develop the same MN degeneration that is characteristic of ALS (Turner and Talbot, 2008). In the past two decades exhaustive efforts have been made to significantly improve the understanding of ALS disease, and at least eight variations of the disease have now been identified (ALS1–ALS8) indicating the complexity of MN degeneration. At present, the G37R, G85R, and G86R transgenic models are considered to be the most reliable and accurate animal models of ALS, where they have been extensively used in understanding the mutation of SOD1 that causes cell death.

Despite the improvement in understanding ALS pathogenesis from the use of transgenic models, there is still a lack of understanding of, or effective treatment for, ALS disease. However, modeling ALS using rodents with an ALS-carrying mutation only represents a subset of the disease, and since ALS is a slowly progressive neurodegenerative disease, modeling ALS using transgenic animal models requires months of study which results in increased cost. To overcome these limitations, the research community has shown keen interest in using iPSC models for ALS disorders. Recent studies have emphasized the involvement of astrocyte dysfunction in the pathogenesis of ALS. One of the standard features of both familial and sporadic ALS is the loss of astrocyte GLT-1, (EAAT2) from the synaptic cleft; regulating synaptic transmission and preventing glutamate excitotoxicity. Supporting the hypothesis of MN degeneration caused by excitotoxicity (Barbeito et al., 2004). Multiple groups have performed ALS iPSC disease modeling from patients with familial and sporadic ALS (Chestkov et al., 2014; Liu et al., 2014). Work by Krencik et al. (2011) in was significant in broadening the tools for ALS research by demonstrating that astrocytes could be generated from human ESCs. When astrocytes generated from hPSCs were transplanted into the lateral ventricles of neonatal mice, they observed that the regional identity specified *in vitro* was retained. Strikingly, the transplanted cells also developed end-feet suggesting that these cells were able to contribute in the formation of the BBB (Krencik and Ullian, 2013). Recently, astrocytes directly reprogrammed from ALS patients carrying *TARDBP* mutations showed abnormalities typical of a TDP-43 proteinopathy, including its cytoplasmic mislocalisation. Imaging of mutant astrocytes revealed that TDP-43 mislocalisation decreased cell survival, suggesting that mutant TDP-43 is responsible for astrocyte pathology. Apparently, mutant astrocytes were not toxic when co-cultured with either control or mutant TARDBP MNs (Serio et al., 2013). In two other reports hiPSCs were utilized to study SOD1 mutation in MNs. Kiskinis et al. (2014) used high-resolution RNA sequencing technologies to identify the transcriptional and functional changes induced by the SOD1A4V dominant mutation in human MNs and found that the expression of mitochondrial-related (Jiang et al., 2013) and ER-stress-mediated genes were significantly reduced. While the other study by Chen et al. (2014) discovered an autonomous behavior of mutated SOD1 in MNs caused by destabilization of neurofilament subunits.

### Hepatic Encephalopathy (HE)

Hepatic Encephalopathy is another example of how astrocyte dysfunction can cause a neurological disease. HE accompanies both acute and chronic liver failure and is characterized by the accumulation of ammonia in the brain that is detoxified by astrocytic GS, resulting in increased osmotic forces, which then leads to cytotoxic oedema and astrocyte swelling. It seems that altered neurotransmission is responsible for the excessive ammonia levels that lead to the cognitive and motor impairments seen in patients with HE. In the brain of HE patients, neurons appear to be morphologically normal, but astrocytes show signs of Alzheimer type II degeneration, i.e., nuclear enlargement, prominent nuclei, chromatin changes, and neurotransmitter receptor alteration (Felipo and Butterworth, 2002; Butterworth, 2010). There have been some animal studies evaluating BBB integrity in acute liver failure, but there has been less research on this subject conducted on humans. To our knowledge, Chen et al. (2012) evaluated the hepatoprotective property of 3-genes iPSC transplantation in a carbon tetrachloride (CCl4)-induced AHF model in mice. Their findings revealed that 3-genes iPSC-based therapy not only improved hepatic functions and animal survival, but also improved CCl4-induced HE. After Chen’s group (Chen et al., 2012), there have been only limited reports addressing the functionality of hepatic cells derived from hiPSCs/hPSCs (Takayama, 2015).

### Alzheimer’s Disease (AD)

Alzheimer’s disease is characterized by the progressive deterioration of cognitive functions such as memory and mental processing. Most cases of AD are sporadic, but about 1–2% are genetically linked with the early onset (EOAD) of dementia. There are two major histopathological hallmarks in the brain of AD patients, the deposition of extracellular SPs composed of the Aβ peptide, and neurofibrillary tangles, which are intracellular inclusions of hyperphosphorylated tau protein in selective regions of the brain (Sidoryk-Wegrzynowicz et al., 2011). SPs are deposits of extracellular Aβ protein derived from Aβ42, a peptide fragment of 42 amino acid residues derived from the sequential step of proteolytic processing of amyloid precursor protein by β and γ secretase. As the disease progresses, synaptic loss and neuronal death become prominent, which consequently lead to the shrinkage of the brain.

The best-studied risk factor for sporadic, LOAD is the presence of ApoE (APOE ε4), a protein involved in lipid metabolism. The presence of one APOE ε4 allele increases the risk of LOAD by threefold while two alleles of APOE ε4 can increase the risk for late-onset AD by 12-fold. Multiple studies from various populations have confirmed the increase in LOAD is caused by the presence of an APOE-ε4 allele. In contrast, APOE-ε2, another variation of this allele, confers protection against developing AD. A recent finding suggests that astrocytes play a significant role in the clearance of the Aβ peptide thus preventing the formation of plaques in the healthy brain (Wyss-Coray et al., 2003; Li et al., 2011). The precise mechanism by which astrocytes recognize and degrade Aβ is not known, but ApoE which is primarily expressed in astrocytes and to a lesser extent in microglia has been proposed to be responsible for this cellular action (Koistinaho et al., 2004; Castellano et al., 2011). The importance of APOE ε4 in AD has been well established, although further studies are needed to understand the exact molecular mechanism that leads to an increased susceptibility for AD.

Another significant aspect of AD pathogenesis is the interaction of microglia and astrocytes through the production of neurotoxic molecules. The role of astrocytes in inflammatory processes is complicated to address, though activated astrocytes are capable of phagocytosis. Activated astrocytes are characterized by hypertrophic somata in neurodegenerative diseases. The process of astrocyte activation results in reactive gliosis, which appears in late stage AD. Reactive astrogliosis in AD can be triggered by various elements, including damaged neuronal signaling and extracellular deposition of the β-amyloid peptide. Once substantial accumulation occurs, astrocytes themselves undergo apoptosis resulting in the formation of amyloid plaques positive for GFAP and S100 β. Recently, iPSC-derived neurons from different patients were found to show different accumulation of Aβ oligomers in AD models (Israel et al., 2012; Kondo et al., 2013) Therefore the use of iPSC-derived AD model can act as a testing platform for the optimal pharmacological regimen.

### Neuromyelitis Optica (NMO)

Neuromyelitis Optica is a primary astrocytopathy disease affecting the CNS. NMO was first described in the 19th century and was long considered to be a variant of multiple sclerosis. Pathological descriptions such as the frequent occurrence of necrosis with cavitation are used to distinguish NMO from multiple sclerosis (Marignier et al., 2010). The disease is commonly associated with diffuse cerebral white matter lesions that resemble acute disseminated encephalomyelitis, and severe demyelination affecting the optic nerve and spinal cord. The evidence of this disease reveals the loss of neurons and astrocytic damage (Wingerchuk et al., 2007). AQP-4 is one of the most valuable tools for the diagnosis of NMO (Lennon et al., 2004), and a novel potential therapeutic approach using a recombinant monoclonal anti-AQP4 antibody that selectively inhibits NMO-IgG binding to AQP-4 has been proposed (Tradtrantip et al., 2012). This study demonstrates a direct way in understanding the pathogenesis of autoimmune disease of NMO in mouse models.

Indeed, several studies have highlighted the relevance of stem cells derived astrocytes in disease modeling. Some examples are mentioned below (**Table 5**).

**Table 5:** Use of human astrocytes generated from pluripotent stem cells for disease modeling and pathology research.

Disorder	Phenotypes	Culture methods	Potential applications	Reference
Amyotrophic lateral sclerosis (ALS)	Down regulation of VAPB expression, failure of motor neurons	By either direct reprogramming or iPSC-derived NPCs	Generated functional astroglia from human induced pluripotent stem cells (iPSCs) carrying an ALS-causing TDP-43 mutation	Serio et al., 2013
			Human iPSC-derived astrocytes showed an increase in Cx43 protein,	Almad et al., 2016
Rett syndrome (RTT)	Fewer synapses, reduced dendritic spine density, and soma size	iPSC-derived NPCs	–RTT-iPSCs showed the recapitulation of RTT phenotypes. –MeCP2 seems to have an essential function in astrocytes	Dajani et al., 2013
Alzheimer disease (AD)	Intracellular accumulation of Aβ, increased ROS production, ER stress	Embryoid body formation by dual smad molecules		Kondo et al., 2013
Huntington disorder	Trinucleotide repeat expansion (CAG) in exon 1 of huntington (HTT)	iPSC-derived NPCs	–Generated adult form of HD (F-HD-iPSCs). –Identified a cellular vacuolation phenotype similar to HD patients.	Juopperi et al., 2012


## Conclusion

Astrocytes are fundamentally involved in various neurological diseases as a consequence of either loss or gain of astrocyte function, and it is now clear that these disorders arise from a complex combination of abnormalities in either neurons, glial cells or immune cells. Recent data suggests that early stages of neurodegenerative disease, for example, are associated with loss of synaptic connectivity, imbalances in neurotransmitter homeostasis, leading to neuronal death probably through increased excitotoxicity in later stages. In the end-stage of neurodegeneration, astrocytes become activated and contribute to neuroinflammatory components of neurodegeneration. Therefore to understand the principles of neurodegeneration and its biology, hPSC-derived neuroglia could serve as a promising tool for creating *in vivo*-like cellular models for dementia and neurological disorders such as ALS, AxD where patient’s neuronal tissue is highly inaccessible.

Neural precursors derived from hPSCs represent an attractive tool for the *in vitro* generation of various neural cell types. In this present review, we have performed a side-to-side comparison of existing protocols and evaluated the astrocyte differentiation based on cellular morphology and gene expression analyses from the human PSCs. Thus RG-like cells expressing RG markers BLBP, GLAST, vimentin, and GFAP could serve as a robust tool for an efficient astroglial generation. We hope this review has shed light on the current progress of astrocyte research.

## Author Contributions

AC has written the manuscript, HA and JK edited the manuscript, AD and ML approved the manuscript. All authors read and approved the final version.

## Conflict of Interest Statement

The authors declare that the research was conducted in the absence of any commercial or financial relationships that could be construed as a potential conflict of interest.

## Abbreviations

Aβ: amyloid-beta
AD: Alzheimer’s disease
ALS: amyotrophic lateral sclerosis
ApoE: apolipoprotein E
AQP-4: aquaporin-4
AxD: Alexander disease
BBB: blood–brain barrier
BMP: bone morphogen protein
CT-1: Cardiotrophin-1
CNS: central nervous system
CNTF: ciliary neurotrophic factor
DS: down syndrome
EAAT1 and EAAT2: excitatory amino acid transporter1/2
EBs: embryoid body
EOAD: early onset Alzheimer’s disease
FGF-2 or bFGF: fibroblast growth factors
GFAP: glial fibrillary acid protein
GDH: glutamate dehydrogenase
GLT-1: glutamate transporter
GS: glutamine synthase
HE: hepatic encephalopathy
hESCs: human embryonic stem cells
iPSCs: induced pluripotent stem cells
iSVZ: inner SVZ
IL-6: interleukin-6
IPC: intermediate progenitor cells
LOAD: late onset-AD
LIF: leukemia inhibitory factor
MCTs: monocarboxylate transporters
MNs: motor neurons
NMO: neuromyelitis optica
oSVZ: outer SVZ
PSCs: pluripotent stem cells
RGs: radial glial
RGC: retinal ganglion cells
RA: retinoic acid
RF: rosenthal fibers
SPs: senile plaques
SHH: sonic hedgehog
SVZ: subventricular zone
SOD-1: superoxide dismutase
TCA: tricarboxylic acid cycle
